# Supplementary material for: The Small RNA Component of Arabidopsis thaliana Phloem Sap and Its Response to Iron Deficiency
Source: Plants (Basel). 2023 Jul 27;12(15):2782. doi: 10.3390/plants12152782 (PMC10421156; doi:10.3390/plants12152782)
Supplement: Supplementary file 1 [file plants-12-02782-s001.zip › Supplementary Table S1.pdf]

Table S1. List of over- and under-represented tRNA genes in phloem exudates. The relative abundance of transcripts from each tRNA gene was analyzed using tRAX (tRNA Analysis of eXpression) software [46]. The change in abundance of transcripts from each gene, and P-value indicating the significance of the difference are shown.

tRNA	Log2FC	p-value
HisGTG.4	6.98650992	4.60E-111
HisGTG.9	6.96024526	4.66E-105
GluTTC.9	6.94948824	1.33E-78
HisGTG.7	6.87543483	1.24E-103
HisGTG.1	6.81991593	6.10E-103
HisGTG.8	6.77566953	7.11E-106
HisGTG.2	6.7132559	5.78E-95
HisGTG.10	6.42242628	2.31E-65
HisGTG.6	6.36978786	6.28E-141
AspGTC.8	6.34572068	2.37E-46
IleAAT.3	6.33677735	1.83E-37
HisGTG.5	6.22723759	4.21E-75
AlaCGC.1	6.20177499	1.57E-73
HisGTG.3	6.19646384	7.18E-75
GlyTCC.10	6.15415442	2.42E-77
GlyTCC.1	6.1522607	4.08E-78
GlyTCC.8	6.14708116	4.79E-78
GlyTCC.5	6.14495001	3.01E-78
GlyTCC.3	6.13553241	5.03E-78
GlyTCC.7	6.13317613	1.01E-77
GlyTCC.11	6.13135921	9.69E-78
GlyTCC.9	6.1272222	1.94E-77
GlyTCC.6	6.1212807	3.28E-77
GlyTCC.2	6.11489357	1.87E-77
AspGTC.26	6.05150501	3.49E-42
GlyTCC.4	6.04619767	5.44E-76

GlyTCC.12	6.02350842	1.34E-75
AspGTC.15	6.00052401	3.55E-41
AspGTC.16	5.94041344	3.11E-39
AlaCGC.5	5.91293411	1.62E-78
AspGTC.12	5.91234304	6.31E-41
AspGTC.25	5.85537807	9.70E-42
LeuTAG.8	5.82983282	1.82E-86
AspGTC.2	5.81021918	2.80E-41
AspGTC.14	5.80442766	2.58E-38
AspGTC.1	5.77664306	3.46E-42
AspGTC.7	5.72386023	3.64E-43
AspGTC.23	5.7097494	2.32E-36
AspGTC.4	5.70643054	3.53E-39
AlaCGC.6	5.68477835	2.99E-73
AlaCGC.2	5.63495622	9.56E-73
AspGTC.20	5.62027937	3.87E-40
AspGTC.10	5.61825275	7.51E-39
AspGTC.9	5.61159674	6.52E-39
ThrAGT.6	5.6088728	3.73E-60
AspGTC.17	5.58880097	8.27E-37
AlaCGC.4	5.58679326	3.29E-59
AspGTC.19	5.49897012	1.01E-34
UndUND.1	5.49580931	3.00E-71
CysGCA.1	5.49211083	4.76E-30
AspGTC.3	5.41524284	4.22E-38
AspGTC.11	5.3926389	4.55E-39
AspGTC.21	5.33098462	3.15E-35
AspGTC.24	5.26805293	1.93E-36
AspGTC.13	5.2362274	3.29E-83
GluTTC.8	5.16726278	3.80E-32

GluTTC.3	5.16537294	2.99E-32
GluTTC.7	4.94691516	3.82E-32
AspGTC.6	4.89238957	4.02E-29
AspGTC.18	4.84920366	3.80E-29
LysTTT.3	4.58532073	2.31E-09
ArgCCG.4	4.51599686	4.31E-17
SerGCT.5	4.37382497	2.65E-85
MetCAT.8	4.16984485	7.89E-25
ValCAC.2	4.13166521	1.20E-64
SerGCT.2	4.10476521	2.62E-62
SerTGA.8	3.74867912	1.07E-23
ArgCCG.5	3.73237341	1.40E-12
ArgCCT.5	3.59699714	1.77E-11
ArgCCG.3	3.57933706	2.74E-11
GluTTC.2	3.55274225	4.91E-14
GluTTC.13	3.53772968	3.87E-14
GluTTC.12	3.53480368	4.96E-14
GluTTC.10	3.53086605	6.14E-14
GluTTC.14	3.53034297	6.31E-14
LysCTT.14	3.51558126	0.000152631
AsnGTT.6	3.42394115	2.21E-28
SerGGA.1	3.39994488	5.52E-31
GluTTC.4	3.39331323	3.70E-13
SerTGA.7	3.28339807	1.87E-17
ThrAGT.3	3.20229986	3.83E-12
CysGCA.13	3.06404092	1.10E-16
ProTGG.37	3.03251366	1.12E-12
MetCAT.13	2.99282751	4.43E-12
TyrGTA.59	2.91726752	1.18E-13

GluCTC.9	2.8585866	4.45E-13
TyrGTA.68	2.81253293	1.78E-13
GluCTC.8	2.81053873	1.58E-12
ThrAGT.4	2.79267776	1.16E-11
ThrAGT.1	2.78313246	3.30E-09
GlyGCC.8	2.72766695	4.08E-10
ThrAGT.5	2.71109817	9.39E-06
GlyGCC.3	2.6607128	5.94E-07
ArgTCG.5	2.58711076	2.59E-14
AlaAGC.1	2.53587803	3.25E-17
ArgTCG.4	2.51182247	4.70E-12
AlaAGC.8	2.49285456	7.05E-15
ArgTCG.3	2.47909446	4.95E-12
TyrGTA.57	2.46303412	9.30E-06
AlaAGC.15	2.43544405	3.77E-14
AlaAGC.12	2.41020405	1.65E-14
AlaAGC.13	2.40994917	3.14E-12
AlaAGC.7	2.40554642	3.46E-14
AlaAGC.10	2.39918258	2.53E-15
GlyACC.1	2.39820095	2.36E-05
AlaAGC.14	2.38200011	6.54E-14
CysGCA.4	2.37257076	6.06E-07
AlaAGC.11	2.36729501	6.25E-13
AlaAGC.5	2.36665038	6.79E-14
AlaAGC.6	2.36502814	2.66E-14
AlaAGC.2	2.36198026	1.29E-14
IleAAT.2	2.35509832	0.026570781
AlaAGC.3	2.3529709	1.13E-11
GlyGCC.6	2.3459348	3.14E-07
AlaAGC.9	2.32573927	1.59E-12

MetCAT.14	2.29927455	1.59E-10
AlaAGC.16	2.28335495	1.38E-11
GlnTTG.5	2.28229775	0.000776722
TrpCCA.6	2.24450807	1.25E-15
ArgTCG.6	2.20022152	1.30E-07
ThrAGT.7	2.15021521	0.003504094
SerTGA.1	2.1461146	9.54E-07
MetCAT.10	2.1235351	0.020583544
GluTTC.6	2.09085309	3.17E-07
GlyGCC.9	1.95362508	0.003717233
GlyGCC.14	1.92896642	0.007257673
AlaTGC.5	1.92587413	3.22E-06
AlaTGC.1	1.91089342	3.16E-05
AlaTGC.4	1.87714899	4.47E-06
GlyGCC.7	1.86254066	7.15E-05
AlaTGC.2	1.8619637	7.86E-06
ArgCCT.6	1.85284822	0.006515685
AlaTGC.6	1.84634006	3.28E-06
AlaAGC.4	1.83681124	4.55E-05
AlaTGC.9	1.83447338	6.92E-05
AlaTGC.7	1.7964661	8.08E-05
CysGCA.3	1.78349772	0.019603472
GlyGCC.1	1.76476992	0.001290906
ArgCCT.8	1.73949905	0.001556018
PheGAA.5	1.73144786	0.000376184
IleAAT.8	1.71054396	0.013619012
ThrAGT.9	1.68504309	0.012934559
GluCTC.11	1.6544612	0.0019239
ArgTCG.2	1.65284991	0.001380552

GluCTC.10	1.62054278	0.002922135
GluCTC.2	1.61762714	0.003064918
TyrGTA.66	1.60510741	0.009373644
GluCTC.12	1.59085109	0.003827711
GluCTC.3	1.58886835	0.004357549
AspGTC.22	1.58745957	0.035712591
GluCTC.13	1.56434792	0.004921427
GluCTC.6	1.56344225	0.009086004
GluCTC.4	1.56245248	0.006453426
GluCTC.1	1.56242464	0.008209847
GluCTC.7	1.55746597	0.005320641
IleAAT.10	1.55366081	0.037927982
AlaTGC.8	1.51415156	0.003405372
AlaCGC.3	1.47755351	0.001974717
ValAAC.15	-1.4370146	0.015428728
ProAGG.3	-1.5313939	0.049895835
GlnCTG.3	-1.5412868	0.00962104
TrpCCA.3	-1.5694036	0.00193786
GlnCTG.4	-1.5697293	0.0026138
GlyGCC.10	-1.5803336	0.024889627
ProTGG.11	-1.588416	0.018344298
LysTTT.14	-1.6268876	0.028588268
ThrCGT.4	-1.6341425	0.014260698
TyrGTA.74	-1.6749532	0.015759774
ProAGG.12	-1.6887309	0.017083218
TyrGTA.70	-1.749457	0.007472825
SerTGA.4	-1.7514851	0.005140779
GlyGCC.24	-1.7626465	0.006385935
SerTGA.3	-1.7873572	0.007452487

GlnCTG.6	-1.7940748	0.000603946
TyrGTA.67	-1.8681415	0.010276463
LeuTAG.4	-1.8817703	0.000152265
GlnCTG.8	-1.8930709	3.64E-05
TyrGTA.76	-1.919218	0.01822488
LeuTAG.9	-1.9246966	0.002136997
ThrTGT.5	-1.9287645	2.28E-06
GlyGCC.18	-1.9326508	0.000102931
CysGCA.10	-1.9548849	0.004720772
AsnGTT.9	-2.0068904	0.000850354
TyrGTA.73	-2.0525028	3.49E-06
LeuTAG.1	-2.0708495	0.000157817
LeuTAG.6	-2.1025238	0.001487155
TyrGTA.61	-2.173831	0.000103571
LeuTAG.2	-2.1769323	1.24E-05
SerAGA.22	-2.1820661	1.05E-16
SerAGA.37	-2.2020603	1.64E-16
LeuTAG.5	-2.2060086	4.58E-07
TyrGTA.43	-2.2365569	0.000363968
SerGCT.9	-2.2592002	0.000569855
ThrTGT.3	-2.3097554	3.88E-06
CysGCA.9	-2.3180854	0.000443663
GlnCTG.1	-2.3704796	0.000370386
TyrGTA.63	-2.4731323	1.32E-07
LeuTAG.10	-2.5233964	6.61E-12
TyrGTA.75	-2.5309322	3.96E-07
TyrGTA.69	-2.5763964	5.98E-05
ProAGG.11	-2.5845441	5.71E-08
TyrGTA.11	-2.6329568	3.56E-12

TyrGTA.34	-2.6434406	1.65E-11
TyrGTA.72	-2.6950874	1.56E-05
GlnTTG.7	-2.7076926	2.01E-09
TyrGTA.62	-2.762859	3.37E-11
GlnTTG.6	-2.7660481	1.52E-06
TyrGTA.8	-2.7910597	6.10E-12
TyrGTA.2	-2.8106138	3.56E-12
TyrGTA.37	-2.8243972	1.73E-08
ProTGG.33	-2.8254844	6.26E-10
TyrGTA.7	-2.8348143	1.82E-13
TyrGTA.39	-2.8556549	7.92E-14
ProTGG.8	-2.8774302	3.41E-05
TyrGTA.9	-2.8779366	1.60E-14
TyrGTA.15	-2.882377	7.65E-12
TrpCCA.1	-2.8902696	1.78E-12
TyrGTA.3	-2.8905342	5.16E-14
TyrGTA.1	-2.8981309	2.94E-14
TyrGTA.41	-2.9025502	6.62E-15
TyrGTA.13	-2.9028126	3.45E-16
TyrGTA.5	-2.9077733	2.75E-14
TyrGTA.12	-2.9151576	1.11E-14
TyrGTA.71	-2.9157312	2.53E-09
TyrGTA.38	-2.9280235	2.53E-13
TyrGTA.23	-2.9291168	2.34E-15
TyrGTA.6	-2.9306427	4.01E-15
TyrGTA.4	-2.9746541	9.02E-17
TyrGTA.27	-2.9956745	9.80E-13
TyrGTA.35	-3.0265575	1.07E-13
TyrGTA.14	-3.0326968	3.60E-16
TyrGTA.45	-3.0468612	4.81E-11

TyrGTA.31	-3.0539298	3.64E-11
TyrGTA.42	-3.0672119	1.23E-12
GlnTTG.2	-3.0708049	1.67E-27
TyrGTA.22	-3.0782546	5.52E-13
SerCGA.4	-3.0951632	1.22E-16
TyrGTA.49	-3.0956747	2.22E-13
TyrGTA.10	-3.0983353	1.74E-16
LeuCAG.2	-3.1166309	2.03E-13
TyrGTA.53	-3.1271198	1.29E-15
TyrGTA.16	-3.1413707	8.29E-19
TyrGTA.18	-3.1480279	2.23E-11
TyrGTA.19	-3.159127	1.20E-15
TyrGTA.64	-3.1734873	3.96E-17
TyrGTA.55	-3.1839708	1.31E-15
TyrGTA.44	-3.1985162	1.42E-16
TyrGTA.51	-3.1998168	1.20E-16
GlnTTG.1	-3.2022469	1.56E-25
TyrGTA.29	-3.2080974	8.15E-18
TyrGTA.56	-3.2192909	9.77E-20
TyrGTA.52	-3.2224924	1.36E-18
GlnTTG.8	-3.2301677	3.41E-28
TyrGTA.46	-3.238446	2.67E-14
TyrGTA.20	-3.2744272	5.51E-18
TyrGTA.33	-3.2785022	3.69E-13
GlnTTG.3	-3.2959507	4.63E-26
TyrGTA.50	-3.3487706	3.01E-18
TyrGTA.24	-3.3595352	9.09E-23
TyrGTA.48	-3.4840479	1.19E-17
TyrGTA.47	-3.5107088	3.24E-20
TyrGTA.25	-3.5237501	1.11E-17

GlnTTG.4	-3.5395435	6.33E-18
TyrGTA.54	-3.5412174	2.08E-17
TyrGTA.28	-3.573423	1.10E-23
TyrGTA.32	-3.5812548	4.61E-23
ArgACG.7	-3.5991154	0.000969295
ProAGG.1<br>0	-4.0960847	6.91E-37
