# Supplementary figures and images for: The Small RNA Component of Arabidopsis thaliana Phloem Sap and Its Response to Iron Deficiency
Source: Plants (Basel). 2023 Jul 27;12(15):2782. doi: 10.3390/plants12152782 (PMC10421156; doi:10.3390/plants12152782)

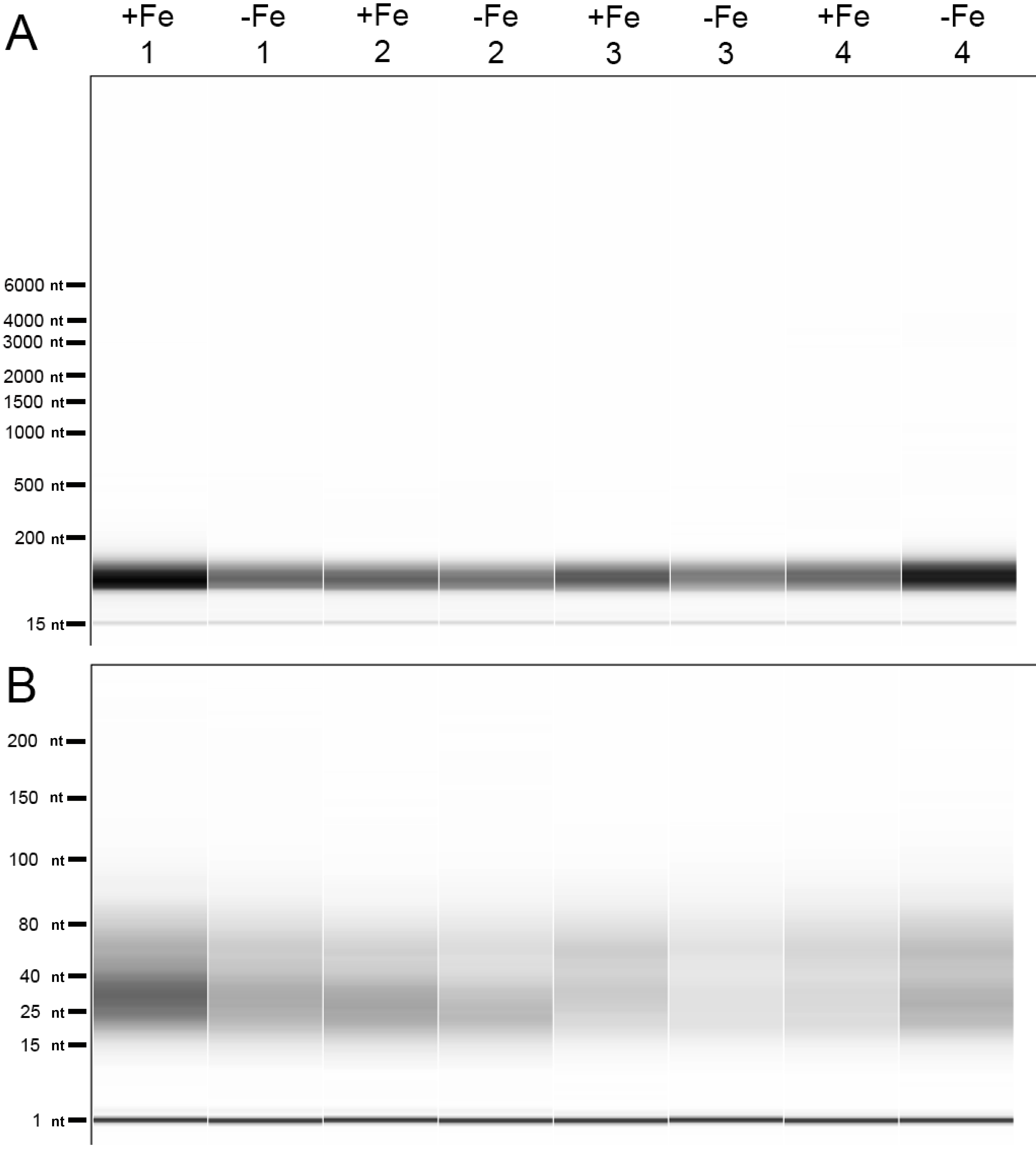

Supplement: Supplementary file 1 [file plants-12-02782-s001.zip › Supplementary_Figure S1.tif]

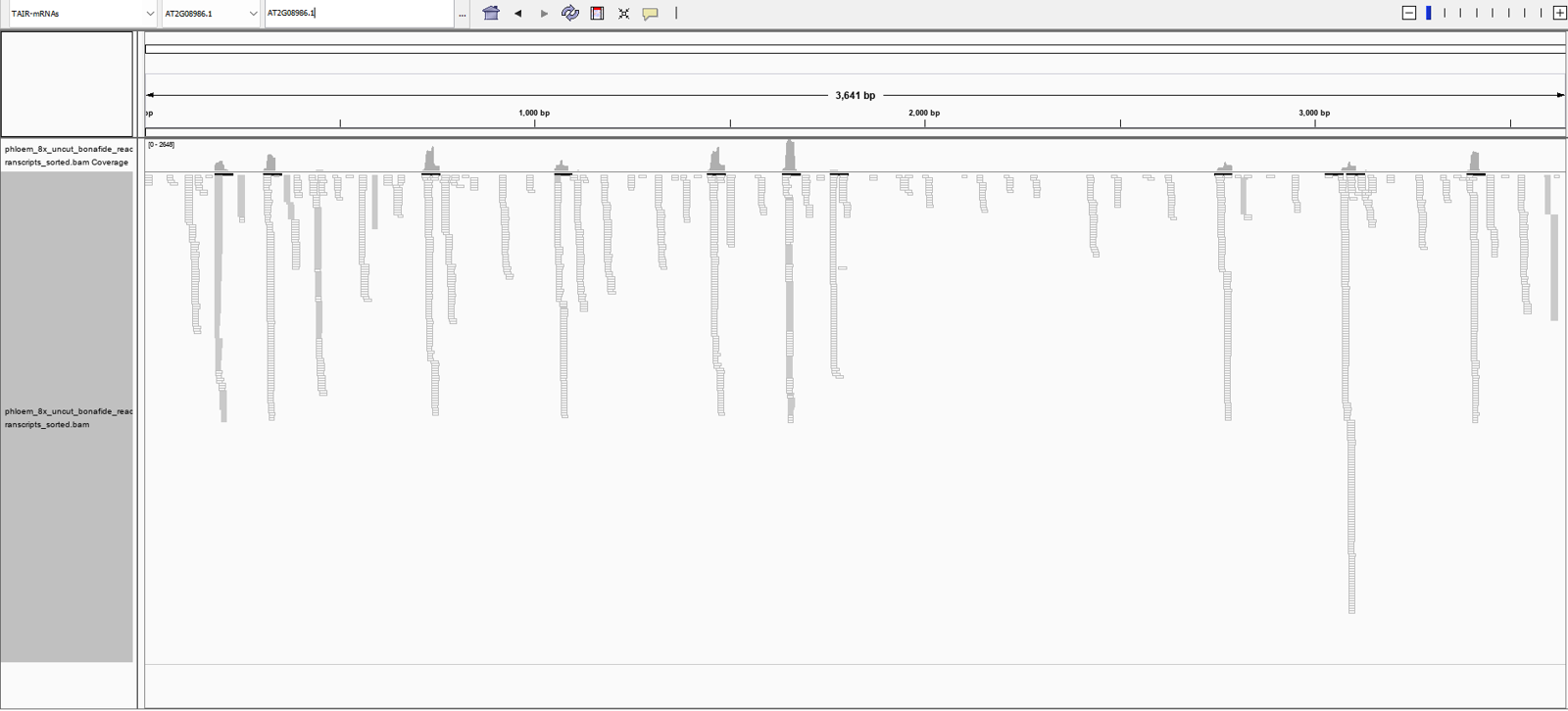

Supplement: Supplementary file 1 [file plants-12-02782-s001.zip › Supplementary_Figure S3.tif]

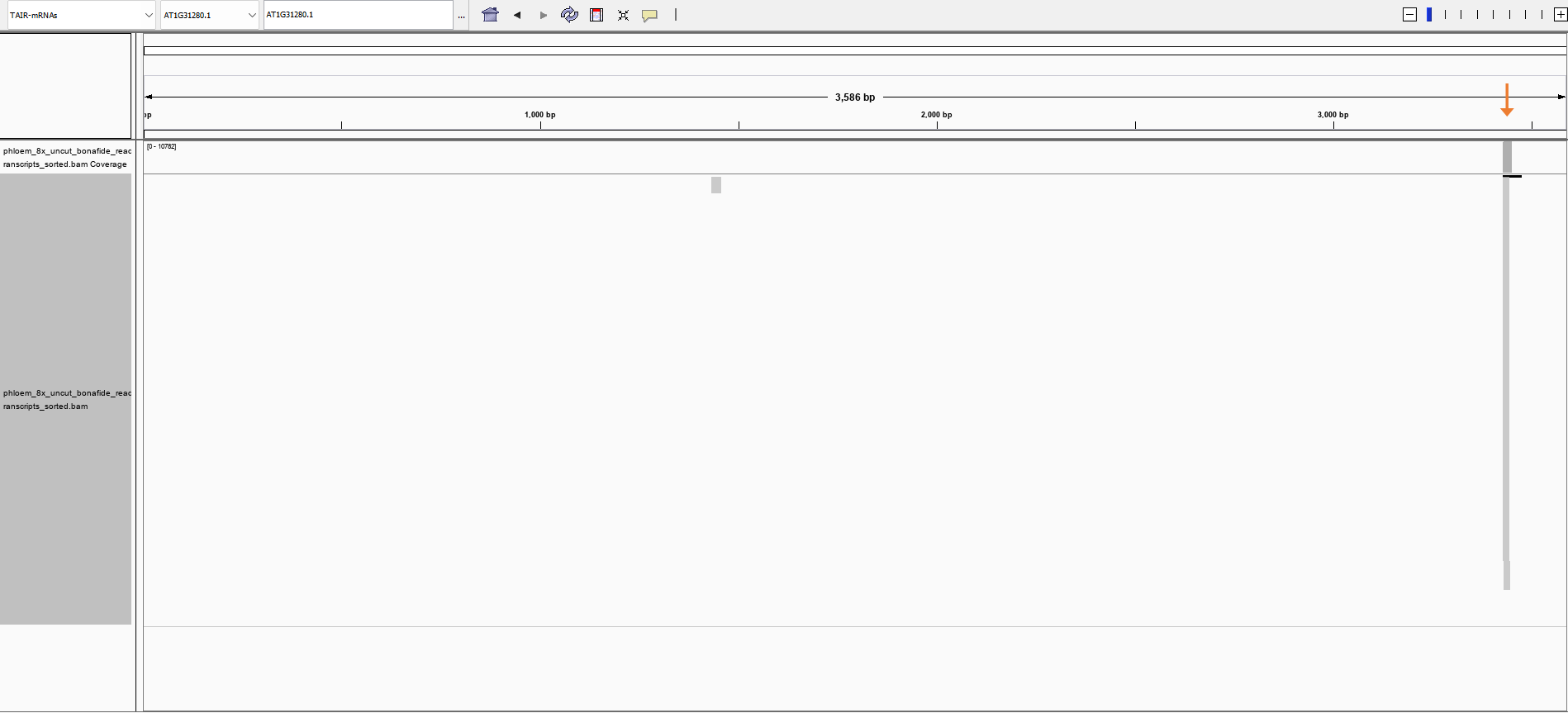

Supplement: Supplementary file 1 [file plants-12-02782-s001.zip › Supplementary_Figure S4.tif]
